# Monocyte chemoattractant protein 1 as a potential biomarker for immune checkpoint inhibitor‐associated neurotoxicity

**DOI:** 10.1002/cam4.5695

**Published:** 2023-02-16

**Authors:** Nora Möhn, Susann Mahjoub, Laura Duzzi, Emily Narten, Lea Grote‐Levi, Gudrun Körner, Tabea Seeliger, Gernot Beutel, Benjamin‐Alexander Bollmann, Thomas Wirth, André Huss, Hayrettin Tumani, Imke Grimmelmann, Ralf Gutzmer, Philipp Ivanyi, Thomas Skripuletz, ICOG‐CCCH (Immune Cooperative Oncology Group; Comprehensive Cancer Center Hannover)

**Affiliations:** ^1^ Department of Neurology Hannover Medical School Hannover Germany; ^2^ Department of Hematology, Hemostasis, Oncology and Stem Cell Transplantation Hannover Medical School Hannover Germany; ^3^ Department of Pneumology Hannover Medical School Hannover Germany; ^4^ Department of Gastroenterology Hannover Medical School Hannover Germany; ^5^ Department of Neurology University Hospital Ulm Ulm Germany; ^6^ Skin‐Cancer‐Center Hannover Medical School Hannover Germany; ^7^ Department of Dermatology Venerology, Allergy and Phlebology Hannover Medical School Minden Germany

**Keywords:** biomarker, immune‐related adverse events, immunotherapy, neurotoxicity, serum neurofilament light chains

## Abstract

**Background:**

Oncological patients can benefit substantially from treatment with immune checkpoint inhibitors (ICI). However, there is a growing awareness of immune‐related adverse events (irAE). Especially ICI‐mediated neurological adverse events (nAE(+)), are tough to diagnose and biomarkers to identify patients at risk are missing.

**Methods:**

A prospective register with prespecified examinations was established for ICI treated patients in December 2019. At the time of data cut‐off, 110 patients were enrolled and completed the clinical protocol. Herein, cytokines and serum neurofilament light chain (sNFL) from 21 patients were analyzed.

**Results:**

nAE of any grade were observed in 31% of the patients (*n* = 34/110). In nAE(+) patients a significant increase in sNFL concentrations over time was observed. Patients with higher‐grade nAE had significantly elevated serum‐concentrations of monocyte chemoattractant protein 1 (MCP‐1) and brain‐derived neurotrophic factor (BDNF) at baseline compared to individuals without any nAE (*p* < 0.01 and *p* < 0.05).

**Conclusion:**

Here, we identified nAE to occur more frequently than previously reported. Increase of sNFL during nAE confirms the clinical diagnosis of neurotoxicity and might be a suitable marker for neuronal damage associated with ICI therapy. Furthermore, MCP‐1 and BDNF are potentially the first clinical‐class nAE predictors for patients under ICI therapy.

## Graphical abstract

Oncological patients can benefit substantially from treatment with immune checkpoint inhibitors. However, there is a growing awareness of immune‐related adverse events, especially neurological adverse events (nAE). MCP‐1 and BDNF are potentially the first clinical‐class nAE predictors for patients under immune checkpoint inhibitor therapy.

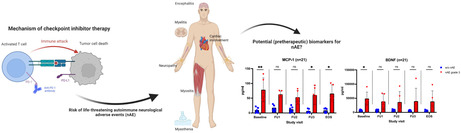

## 1 INTRODUCTION

Treatment with immune checkpoint inhibitors (ICI) has revolutionized the therapy of metastatic cancer and is unequivocally one of the most important developments in cancer treatment over the past decade.^1^ Since the approval of ipilimumab for treatment of metastatic melanoma in 2011, seven other checkpoint inhibitors were approved for the treatment of several other entities, for example, renal cell carcinoma (RCC), non‐small cell lung cancer (NSCLC), urothelial carcinoma (UCC), or head and neck squamous cell carcinoma.^2^, ^3^, ^4^, ^5^, ^6^, ^7^, ^8^, ^9^

Often, tumor cells are able to escape host's cancer specific T‐cells response by aberrant activation of immunological checkpoint‐pathways. This immune evasion strategy allows cancer cell to escape T‐cell mediated immunotoxicity.^5^ In this context, immune checkpoint inhibitors, blocking immunological escape pathways could increase T‐cell activity and ultimately enable T‐cell mediated tumor response. The typical ICIs used in the clinical setting are antibodies that target the most studied immune checkpoints, namely cytotoxic T‐lymphocyte‐associated protein 4 (CTLA‐4) and programmed cell death protein 1(PD‐1) or its ligand PD‐L1.^10^, ^11^, ^12^

However, the often outstanding long anti‐tumor efficacy of ICI therapy may be accompanied by relevant side effects. The pronounced activation of the immune system is associated with a new range of side effects called “immune‐related adverse events” (irAEs). They may closely resemble autoimmune diseases and can lead to permanent damage and even death. IrAEs can occur in almost every organ, especially skin, lung, gastrointestinal tract, endocrine system, pancreas, but also the nervous system.^13^ While autoimmune dermatitis, colitis, hypophysitis, and other endocrinopathies are frequently described, neurological adverse events (nAE) can be more difficult to recognize.^14^ However, they especially can lead to long‐term sequelae and may be fatal or at least treatment‐limiting if handled too late.^15^ The highest incidences of nAE appear in combination therapy with anti‐CTLA‐4 and anti‐PD1 antibodies.^16^ While irAEs in general are most commonly described during anti‐CTLA‐4 therapies,^17^ nAE occur more often with anti‐PD‐1 therapy when focusing solely on monotherapies.^17^ The underlying pathogenesis of neurotoxicity has not been fully understood yet. Many nAEs are described only in case reports or small case studies and once they appear, they are predominantly treated with corticosteroids, similar to other irAE. Among all autoimmune neurological adverse events predominantly myasthenia gravis, peripheral neuropathies, Guillan–Barré–syndrome, encephalitis, and meningitis have been reported.^18^

To determine the prevalence, the incidence and the characteristics of neurotoxicity in patients receiving ICI, we designed a prospective monocentric cohort study. Besides clinical examination, sequentially collected blood samples were investigated to detect potential biomarkers for nAE during ICI therapy and to develop a pretherapeutic risk stratification.

## 2 PATIENTS AND METHODS

### 2.1 Study design and patient selection

Since December 2019, a prospective cohort study has been established at the Hannover Medical School (MHH). Herein, the immune‐oncology‐working group (ICOG), an interdisciplinary cooperation among the Department of Neurology, the Skin‐Cancer‐Center, the Department of Hematology, Hemostasis, Oncology and Stem Cell Transplantation, the Department of Pneumology, and the Department of Gastroenterology at Hannover Medical School are in charge for all included patients (supplemental Figure S1). All oncological patients, from mentioned departments, with ICI inhibition were prospectively included in this cohort, after patients gave written informed consent. The cohort included patients with different cancer entities which were naive for ICI. Patients must receive at least one dosing of ICI. Administered ICI were: nivolumab, nivolumab/ipilimumab, pembrolizumab, atezolizumab, avelumab, cemiplimab, and treatment followed the summary of product characteristics (SmPC). Clinical descriptive data of the study cohort included age, sex, underlying oncological diseases, type and dosage of ICI, therapy cycles and concomitant diseases as well as tumor outcome.

Due to the study protocol, each patient was examined at five initially pre‐defined time points (supplemental Figure S1): (i) before ICI therapy (baseline); (ii) 3–4 weeks after baseline (Follow up [FU] 1); (iii) 3–4 weeks after FU1 (FU 2); (iv) 3–4 weeks after FU2 (FU 3); (v) 6 months after baseline (end of study visit (EOS)). At baseline and EOS patients received a detailed clinical neurological examination. Furthermore, the inflammatory neuropathy cause and treatment disability score (INCAT) and the inflammatory Rasch‐built overall disability score (I‐RODS) were ascertained at each time point and patients were interviewed regarding possible side effects of ICI treatment. Occurring irAEs and nAE were classified into severity grades ranging from 1 (mild) to 5 (death as a result of adverse events), based on the Common Criteria for Adverse Events (CTCAE) version 6.0. Myopathy was defined as muscle pain in combination with elevated creatine kinase. Blood samples including plasma and serum were collected at baseline and at each follow‐up visit including EOS. They were further processed and examined in order to identify biomarkers for the occurrence of irAEs and especially nAE. In particular, cytokine concentrations and neurofilament levels in the serum of patients were investigated in this project.

The study protocol was approved by the local ethics committee at Hannover Medical School (No. 8685_BO_K2019) following the Declaration of Helsinki (supplemental Figure S2).

### 2.2 Neuroinflammation panels/flow cytometry

LegendPlex assays were utilized to define levels of circulating chemokines and cytokines. Examinations were performed as described in the belonging manual. We used the LegendPlex Human Neuroinflammation Panel 1, a bead‐based ELISA, using fluorescence binding beads appropriate for several flow cytometers by BioLegend (BioLegend, San Diego, CA). This assay allows quantification of 13 cytokines and chemokines involved in neuroinflammation with their maximum detection concentration, respectively: visinin‐like protein 1 (VILIP‐1, max. det. Conc. 200,000 pg/mL), monocyte chemoattractant protein‐1 (CCL2 /MCP‐1, max. det. Conc. 10,000 pg/mL), triggering receptor expressed on myeloid cells 2 (sTREM‐2, max. det. Conc. 25,000 pg/mL), brain‐derived neurotrophic factor (BDNF, max. det. Conc. 10,000 pg/mL), transforming growth factor beta (TGF‐ß1, max. det. Conc. 20,000 pg/mL), vascular endothelial growth factor (VEGF, max. det. Conc. 50,000 pg/mL), Interleukin‐6 (IL‐6, max. det. Conc. 10,000 pg/mL), triggering receptor expressed on myeloid cells 1 (s‐TREM‐1, max. det. Conc. 50,000 pg/mL), nerve growth factor (ß‐NGF, max. det. Conc. 2500 pg/mL), interleukin‐18 (IL‐18, max. det. Conc. 10,000 pg/mL), tumor necrosis factor‐alpha (TNF‐α, max. det. Conc. 10,000 pg/mL), soluble receptor for advanced glycation end products (sRAGE, max. det. Conc. 100,000 pg/mL) and cytokine‐induced neutrophil chemoattractant type‐1 (CX3CL1, max. det. Conc. 400,000 pg/mL). In this assay, the analytes are captured by a specific bead set with appropriate capture antibodies on the surface. The concentration of a particular analyte is determined by using a standard curve generated in the same assay. Standards and samples were analyzed on a BD FACSCanto II Flow cytometer (BD Biosciences, San Jose, CA) with FACSDiva Software version 7. After proper set up the flow cytometer differentiates specific beads for each analyte by size and the specific combination of two fluorochromes. For each analyte at least 300 events were acquired as recommended by the manufacturer. The results were analyzed with the LEGENDplex Data Analysis Software Mac OS version 10.7 (Biolegend, San Diego, CA).

Serum concentrations of the above‐mentioned cytokines (namely VILIP‐1, MCP‐1, sTREM‐2, BDNF, TGF‐ß1, VEGF, IL‐6, s‐TREM‐1, ß‐NGF, IL‐18, TNF‐α, sRAGE, and CX3CL1) were analyzed at multiple time points. All patient groups (i.e., individuals without nAE and with nAE CTCAE grades 1, 2, and 3) were compared.

### 2.3 Analysis of neurofilament serum levels

Serological neurofilament light chain concentrations were analyzed with commercially available kits for the ELLA microfluidic system (Bio‐Techne, Minneapolis, USA) and measurements were performed according to the manufacturer instructions. Intra‐ and inter‐assay variation was determined by a serum control pool measured in quintuplicates and was <10%.

### 2.4 Statistics

Statistical analyses were performed using graphpad prism (version 8). Two‐way ANOVA with Tukey's multiple comparison test was used to compare the cytokine and neurofilament measurements of the different groups. Results with *p* < 0.05 were considered statistically significant.

## 3 RESULTS

### 3.1 Patients' characteristics

Between December 2019 and February 2021 110 patients were included in the study (Table 1), of which 34 patients developed nAE. The median age of the total cohort was 63 (range: 29–87) years. Among the 110 patients, 41 (37%) were female. The clear majority of patients (*n* = 76; 69%) suffered from malignant melanoma. Comparing the subgroups of patients with and without nAE hardly any differences became obvious, although significantly more patients in the nAE subgroup suffered from NSCLC (*p* = 0.0003) (Table 1).

**TABLE 1:** Patient characteristics of the overall cohort and the subgroup with and without nAE.

Parameters	Total cohort (*n* = 110)	With nAE (*n* = 34)	Without nAE (*n* = 76)	*p*‐value
*Age at start of ICI*				
Median (min–max), years	63 (29–87)	63 (29–87)	63 (30–86)	0.486
*Sex*				0.258
Female, *n* (%)	41 (37%)	10 (29%)	31 (41%)	
Male, *n* (%)	69 (63%)	24 (71%)	45 (59%)	
*Underlying tumor disease, n* (%)				
Malignant Melanoma	76 (69%)	21 (61.8%)	55 (72.37%)	0.211
NSCLC	10 (9%)	8 (23.5%)	2 (2.63%)	**0.0003**
Renal cell carcinoma	9 (8%)	2 (5.88%)	7 (9.21%)	0.560
Head and neck tumor	5 (4.5%)	1 (2.94%)	4 (5.26%)	0.593
Squamous cell carcinoma	2 (1.8%)	0	2 (2.63%)	0.344
HCC	1 (1%)	0	1 (1.32%)	0.506
Endocrine carcinoma	1 (1%)	0	1 (1.32%)	0.506
Pleural mesothelioma	1 (1%)	0	1 (1.32%)	0.506
Tonsillar carcinoma	1 (1%)	1 (2.94%)	0	0.136
CUP	1 (1%)	1 (2.94%)	0	0.136
Combination	3 (2.7%)	0	3 (3.95%)	0.244
*ICI used*, *n* (%)				
Nivolumab	61 (55.5%)	15 (44.2%)	46 (60.5%)	0.144
Pembrolizumab	20 (18.2%)	9 (26.5%)	11 (14.5%)	0.089
Nivolumab/Ipilimumab	19 (17.3%)	7 (20.6%)	12 (15.7%)	0.543
Atezolizumab	4 (3.6%)	1 (2.9%)	3 (3.95%)	0.797
Cemiplimab	2 (1.8%)	1 (2.9%)	1 (1.31%)	0.559
Avelumab	1 (0.9%)	0	1 (1.31%)	0.506
Ipilimumab	1 (0.9%)	0	1 (1.31%)	0.506
PD‐1‐antibody + TKI	2 (1.8%)	1 (2.9%)	1 (1.31%)	0.559
*Number of prior therapies, n* (%)				
0	73 (66.4%)	23 (67.7%)	50 (65.8%)	0.851
1	20 (18.2%)	6 (17.7%)	14 (18.4%)	0.923
2	7 (6.4%)	3 (8.8%)	4 (5.3%)	0.484
3	2 (1.8%)	1 (2.9%)	1 (1.3%)	0.559
>3	5 (4.5%)	1 (2.9%)	4 (5.3%)	0.593
N/A	3 (2.7%)	0	3 (3.9%)	0.244
*Comorbidities*, *n* (%)				
None	42 (38.2%)	15 (44.1%)	27 (35.5%)	0.396
Cardiovascular	57 (51.8%)	15 (44.1%)	42 (55.3%)	0.284
Autoimmune	1 (0.9%)	0	1 (1.3%)	0.506
Combination CVSC + COPD	4 (3.6%)	3 (8.8%)	1 (1.3%)	0.152
Other combinations	3 (2.7%)	1 (2.9%)	2 (2.6%)	0.927
N/A	3 (2.7%)	0	3 (3.9%)	0.244
*Specific irAE other than nAE total events, n* (%)	45	8	37	
Thyroiditis/hyperthyroidism	11 (24.5%)	2 (25%)	9 (24.3%)	0.330
Hypophysitis	1 (2.2%)	0	1 (2.7%)	0.506
Dermatitis	3 (6.7%)	3 (37.5%)	0	**0.008**
Mucositis	2 (4.4%)	1 (12.5%)	1 (2.7%)	0.559
Hepatitis	8 (17.8%)	1 (12.5%)	7 (18.9%)	0.246
Vitiligo	2 (4.4%)	1 (12.5%)	1 (2.7%)	0.559
Pancreatitis	2 (4.4%)	0	2 (5.4%)	0.344
Colitis	8 (17.8%)	0	8 (21.6%)	0.050
Autoimmune thrombocytopenia	1 (2.2%)	0	1 (2.7%)	0.506
Nephritis	3 (6.7%)	0	3 (8.1%)	0.244
Arthritis	2 (4.4%)	0	2 (5.4%)	0.344
Hemophagocytic lymphohistiocytosis	1 (2.2%)	0	1 (2.7%)	0.506
Pneumonitis	1 (2.2%)	0	1 (2.7%)	0.506
*Patients with >1 specific irAE including nAE, n* (%)	16 (14.5%)	7 (20.6%)	9 (11.8%)	0.233

Abbreviations: COPD, chronic obstructive pulmonary disease; CUP, cancer of unknown primary; CVSC, cardiovascular; HCC, hepatocellular carcinoma; ICI, immune checkpoint inhibitor; irAE, immune‐related adverse events; N/A, not applicable; nAE, neurological adverse events; NSCLC, non‐small cell lung cancer; TKI, tyrosine kinase inhibitor.

Bold values indicates statistical significance.

PD‐1‐based therapies were predominantly administered (nivolumab (*n* = 61), pembrolizumab (*n* = 20)). Patients with nAE were overall more likely to receive combined ICI with nivolumab and ipilimumab, or therapy with pembrolizumab, although this difference was not significant compared with patients without neurotoxicity (Table 1). The median time from disease diagnosis to the start of immunotherapy was 13.5 months (range: 1–428 months). Immunotherapy was the first medical treatment in 73 of 110 patients (66%). Twenty patients (18%) had previously received systemic therapy, including chemotherapies, immunotherapies other than ICI, tyrosine kinase inhibitors, BRAF inhibitors, and VEGF inhibitors. Eight patients (9.4%) died from the tumor disease during study conduction, while 77 patients (90.6%) were alive at time of last follow‐up EOS. 25 patients (22.7%) discontinued the study early due to any reason (unable to continue therapy, palliative care, or change of treatment center). Among concomitant diseases, 63 patients (57%) had a previous cardiovascular disease such as arterial hypertension or heart failure alone or in combination with other diseases. In four cases, pulmonary disease was present, and two patients suffered from autoimmune disease at baseline; none of the patients suffered from neurological diseases prior to the start of ICI.

### 3.2 Immune‐related adverse events of the total cohort

**FIGURE 1:**
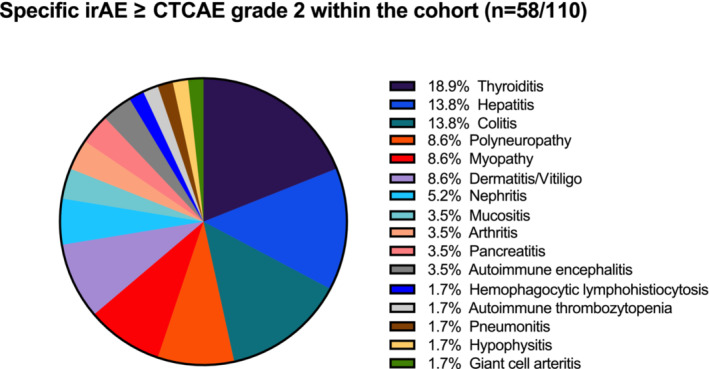
Illustration of specific immune‐related adverse events (irAE) including neurological adverse events (nAE) within the total cohort (*n* = 58/110, 52.7%). Displayed are specific ICI‐induced autoimmune adverse events of ≥CTCAE grade 2 including specific nAE. CTCAE, common criteria terminology for adverse events. Version 6.0 CTCAE was used.

IrAE of any grade occurred in 81% of ICI‐treated patients (*n* = 89/110). In 17 of 110 patients (15%), ICI was ceased due to adverse events, and temporarily interrupted in 8% of the patients. In 16.5% of the patients (*n* = 18/110) hospitalization was necessary. A total of 45 cases of higher‐grade irAE were described, including 37 in the subgroup without nAE and eight in patients who also developed nAE (Table 1). AE of CTCAE grade 2 or higher are depicted in Figure 1. Among all irAE thyroiditis or hyperthyroidism were most frequent (24.5%) (Table 1). Within the nAE(+) group, however, dermatitis was the most common additional irAE (37.5%) alongside existing nAE, significantly more often compared to the nAE(−) group (*p* = 0.008). Other common specific irAE of the overall cohort were hepatitis (*n* = 8) and colitis (*n* = 8), the latter being detected only in the non‐nAE subgroup. It is important to note that patients with higher grade irAE, often suffered from more than just one autoimmune side effect. More than one specific irAE/nAE occurred in 16 cases, with the most common combination being autoimmune colitis and hepatitis. nAE(+) patients tended to show concomitant other irAE more frequently (20.7%) than nAE(−) patients (11.8%, *n* = 9).

### 3.3 Neurological adverse events within the cohort and characteristics of the patients with cytokine measurements

**FIGURE 2:**
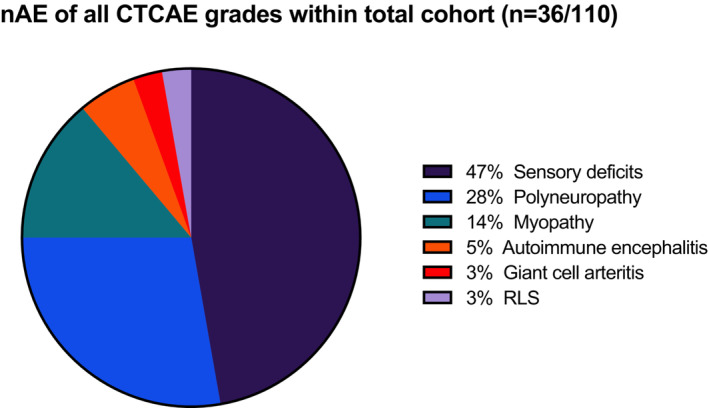
Illustration of neurological adverse events (nAE) of the total cohort (*n* = 36/110, 32.7%). Displayed are neurological adverse events of varying severity (CTCAE grades 1 – 4). Sensory deficits include hypesthesias, dysesthesias, and paresthesias. CTCAE, common criteria terminology for adverse events; RLS, restless‐legs‐syndrome. Version 6.0 CTCAE was used.

A total of 36 nAEs of any grade were identified within the cohort (Figure 2). While 31% (*n* = 34/110) of all patients suffered from nAE, two of them experienced two different nAE, namely autoimmune encephalitis in combination with demyelinating polyneuropathy and sensory deficits combined with reduced muscle strength (Figure 2).

Serum analyses were performed in 19% of all patients (*n* = 21/110) (supplemental Table S1), herein had no nAE. 16 patients suffered from nAE, 5 of grade 1, 6 and 5 of grade 2 and 3, respectively. The median age of this subgroup in whom cytokines were measured was 60 years (range: 29–75 years) with a gender distribution of 7 women (33%) and 14 men (67%). Patients with nAE grade 1 (*n* = 5) had transient paresthesia or hypesthesia of the extremities without manifest polyneuropathy. Among grade 2 nAE sensory polyneuropathy (*n* = 2), myopathy (*n* = 3), lack of strength in combination with balance disorders (*n* = 1), and holocephalic headache with bulbar movement pain (*n* = 1) was reported. The grade 3 nAE group included autoimmune encephalitis (*n* = 2), giant cell arteritis with consecutive visual loss (*n* = 1), and demyelinating polyneuropathy with motor deficits (*n* = 3). In four patient cases ICI therapy was paused or ceased due to irAE.

### 3.4 Patients with higher‐grade neurotoxicity show an increase in sNFL over time

**FIGURE 3:**
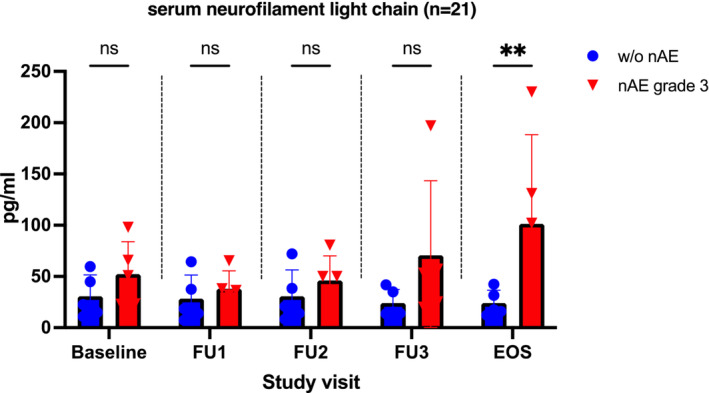
Comparison of neurofilament light chain in serum in patients without nAE and those with nAE grade 3. EOS, end‐of‐study; FU, follow‐up; nAE, neurological adverse events; ns, not significant. ** *p* < 0.01. At each time point/study visit, *n* = 5 patients from each group were studied. None of the patients with nAE showed neurological symptoms at baseline.

Analysis of serum neurofilament light chain (sNFL), which reflects axonal damage in a wide variety of neurological disorders, tends to show increased levels in patients with higher grade nAE with significant differences at last visit (*p* < 0.01) (Figure 3). No significant differences were observed when sNFL concentrations were compared between patients without nAE and lower‐grade nAE (data not shown).

### 3.5 Patients with and without neurotoxicity exhibit differences in cytokine concentrations at baseline

**FIGURE 4:**
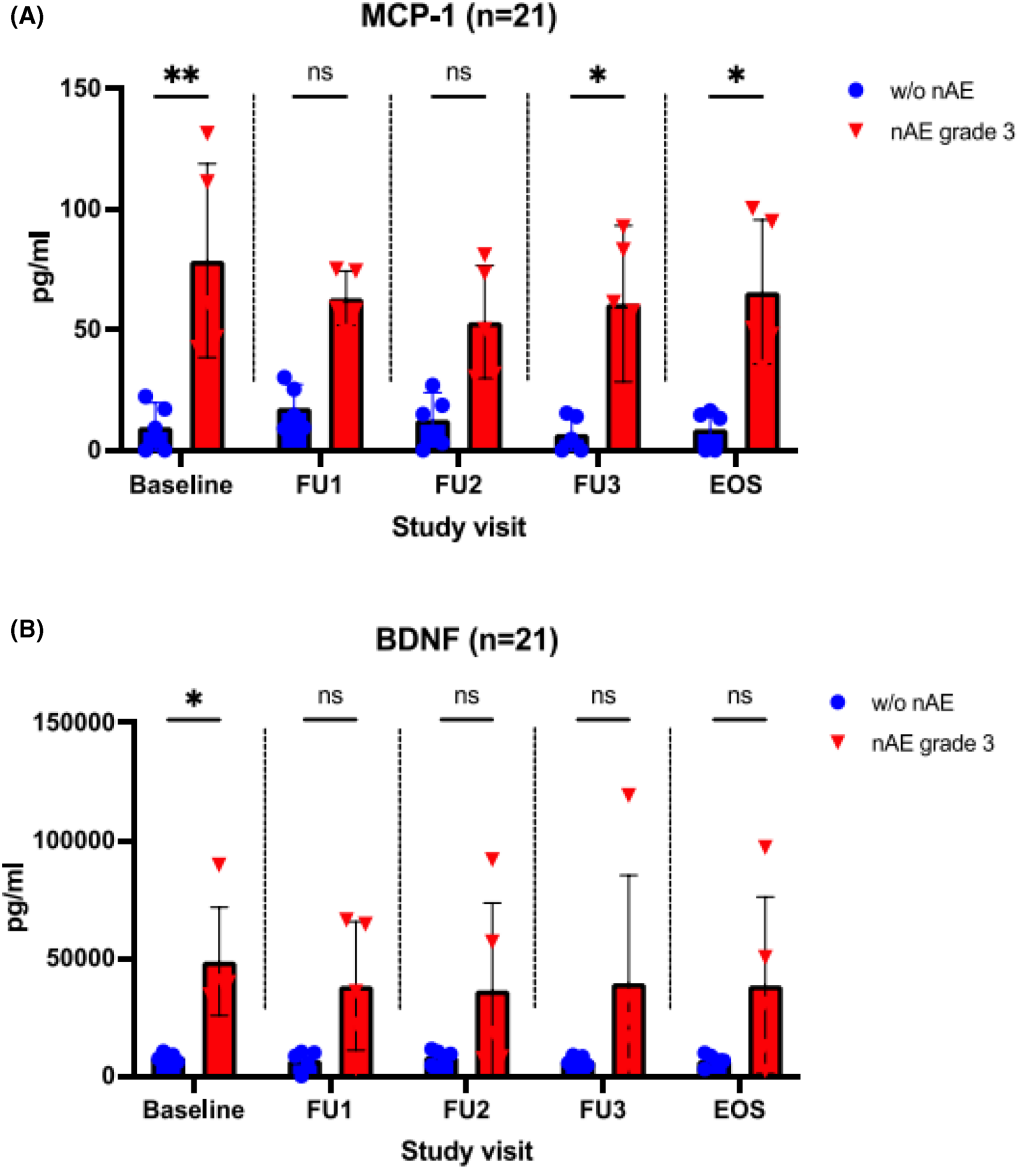
Cytokine measurement in patients with and without ICI‐associated neurotoxicity CTCAE grade 3. (A) Serum monocyte chemoattractant protein 1 (MCP‐1) concentrations, comparison of patients without nAE and those with nAE grade 3. (B) Serum levels of brain derived neurotrophic factor (BDNF), comparison of patients without nAE and those with nAE grade 3. EOS, end‐of‐study; FU, follow‐up; nAE, neurological adverse events; ns, not significant. ** *p* < 0.01, * *p* < 0.05. At each time point/study visit, *n* = 5 patients from each group were studied. None of the patients with nAE showed neurological symptoms at baseline.

Serum concentrations of various cytokines (namely VILIP‐1, MCP‐1, sTREM‐2, BDNF, TGF‐ß1, VEGF, IL‐6, s‐TREM‐1, ß‐NGF, IL‐18, TNF‐α, sRAGE, and CX3CL1) were analyzed at prespecified time points. Patients with nAE ([+]) and without ([−]) (i.e., individuals without nAE and with nAE CTCAE grades 1, 2, and 3) were compared. Herein, in nAE (+) grade 3 patients monocyte chemoattractant protein 1 (MCP‐1) was significantly elevated compared to nAE(−) patients at baseline and 3 and 6 months after therapy initiation. This difference was particularly pronounced at baseline. However, among nAE (+) patients no significant increase in MCP‐1 serum concentration was observed over time (Figure 4A). Significant differences (*p* < 0.05) in serum concentration were found at all measured time points when comparing nAE(+) grade 2 versus nAE(−). Additionally, grade 1 nAE(+) patients showed a significant higher MCP‐1 baseline‐concentration than those without nAE (supplemental Figure S3B). Pooled analysis of all nAE(+) patients compared with nAE(−) patients showed significant differences in MCP‐1 serum concentration at baseline as well as at follow‐up 3 and end‐of‐study visit (Supplemental Figure S3A). For all other cytokines, there were no relevant differences when comparing nAE(−) patients to those with grade 1 or 2 neurotoxicity (data not shown).

Regarding the serum concentration of brain‐derived neurotrophic factor (BDNF), a significant difference at baseline was identified among grade3 nAE(+) versus nAE(−) patients, while neither a relevant difference among the grade 1 and grade 2 nAE(+) subgroups compared to nAE(−) (Supplemental Figure S3C), nor a significant increase within the nAE+ patient group was identified during follow up examinations (Figure 4B). When measuring IL‐6, s‐TREM‐1, or sRAGE, there was a tendency toward increased serum concentrations in patients with higher‐grade nAE, while for all other cytokines examined, no differences could be found in the serum of the different patient groups (Supplemental Figure S4).

## 4 DISCUSSION

Therapy with ICI is standard of care in medical treatment of many cancers and up to date the approvals in oncology are still rising. Nevertheless, knowledge about the etiology and pathogenesis of autoimmune neurological adverse events is limited. Here, we report on a prospective evaluation of nAE, as well as on biomarkers associated with.

Compared to recent literature, the incidence of neurotoxicity during ICI‐ therapy is increased within our cohort (*n* = 36/110, all grades, *n* = 6, grade 3/4).^19^, ^20^ This might most likely result from our administered study protocol wherein patients receive regular monitoring by a neurologist. Ultimately, this reflects the benefits of multi‐professional patients' visits. However, one might speculate, whether or not the clinical relevance of this diagnostic is impacting patients' outcome, since the majority of nAE were low grade and treatment cessation of ICI or significant postponement of therapy were infrequent. None the less, also severe and life‐threatening nAE were identified, herein particular predictive biomarker would be of general interest to rise preventive awareness for those rare, but hard to detect nAE.

Concentration of sNFL differed significantly at 6 month post ICI‐initiation (end of observation period) comparing nAE(−) versus nAE(+) patients (Figure 3.), while clinical presence of nAE showed up prior to 6 months, suggesting that axonal damage has already taken place. This same effect has been shown for several neurological diseases including multiple sclerosis, where NFL is discussed as a useful biomarker for disease activity.^21^, ^22^, ^23^ Thus, the determination of neurofilaments over time might be a useful complementary diagnostic tool for the detection or quantification of neurotoxicity under ICI therapy, rather than for prediction.

To date, little published work can be found on predictive biomarkers for irAEs, especially with regard to nAE. It has been shown that CCL5 could serve as a potential biomarker for development of irAEs. CCL5 levels increase 4 weeks after initiation of nivolumab therapy in NSCLC‐patients who develop irAEs.^24^ Furthermore, lower levels of CXCL9, CXCL10, CXCL11, and CCL19 were associated with irAEs in patients with solid tumors treated with anti‐PD‐(L) 1 therapy.^25^ On the other hand, a high neutrophil‐to‐lymphocyte ratio appeared to predict a lower risk of immune‐mediated toxicities,^26^ but correlated with worse overall survival in multiple ICI‐treated malignancies.^27^ Another postulated marker for the occurrence of irAEs is the prognostic nutrition index (PNI) calculated from serum albumin levels and total lymphocyte count. A PNI of 45 or higher is thought to be associated with an increased risk of irAE.^28^ For individual specific nAE such as autoimmune encephalitis or myasthenia gravis, distinct autoantibodies are occasionally found (anti‐Ma2‐ or anti‐acetylcholine receptor antibodies),^29^, ^30^ although this is certainly the exception. In most cases, patients with ICI‐associated neurotoxicity are antibody‐negative.

Here, we were able to demonstrate that patients with higher grade neurotoxicity showed significantly elevated MCP‐1 serum levels at baseline compared to non‐nAE patients. MCP‐1 (or CC ligand‐2—as it is also referred to) is a chemokine belonging to the CC‐family. As the name implies, it is a potent chemoattractant and activator for monocytes promoting their infiltration into tumors by binding to CC chemokine receptors (CCR)‐2A and 2B.^31^ Through the induction of metalloproteinases (MMP) 2 and 9 expression in cancer cells, it forms the basis for their successful metastasis. Furthermore, it leads to production of angiogenic factors promoting angiogenesis and stimulates cell proliferation and survival.^32^ The MCP‐1‐CCR‐2 axis also contributes to immunological processes. Besides activation and differentiation of monocytes, it directs leukocyte infiltration and proliferation of T cells. Produced by epithelial cells, endothelial cells, smooth muscle cells, macrophages, and fibroblasts, it is one of the most highly expressed chemokines during inflammation.^33^ Within the brain, it has been discussed as a biomarker in acute brain injury as well as in ischemic stroke.^34^, ^35^, ^36^ An association with autoimmune CNS diseases such as multiple sclerosis has been investigated as well. However, no explicit connection could be proven so far, but MS patients treated with interferon‐ß showed a decreased MCP‐1 production from monocytes following T‐cell activation compared to untreated patients.^31^, ^37^ It can be stated that to date the research results attribute a role to MCP‐1—predominantly in CNS inflammation. That is why the cytokine is mainly examined in the CSF. Since neurotoxicity triggered by ICI therapy occurs in the peripheral nervous system in the majority of cases, a possible significance of MCP‐1 in this area of the nervous system is of particular importance. However, only one publication can be found describing an increased concentration of MCP‐1 in the CSF of patients with acute Guillain‐Barré syndrome.^38^ References on the relevance of serum‐MCP‐1 in autoimmune neurological diseases are almost completely lacking. Nevertheless, the characteristics mentioned so far might qualify MCP‐1 as candidate for a suitable predictive biomarker for nAE occurrence. However, further studies with larger patient cohorts seem to be reasonable to verify the present results.

Among the multiple cytokines and chemokines studied, BDNF has emerged as a second potential candidate, for pretherapeutic differentiation of patients with and without neurotoxicity. Yet, there is only a difference at baseline, and this is less pronounced than for MCP‐1 (Figure 4B). BDNF, a member of the neurotrophin family, is translated as pro‐BDNF and cleaved into mature BDNF by endoproteases and metalloproteinases.^39^ It influences serotonergic and dopaminergic neurotransmission through modulation of neuronal differentiation and has an important role in proper growth, development, and plasticity of glutamatergic and GABAergic synapses.^40^ Interestingly, its gene expression is strongly regulated by a wide array of endogenous and exogenous stimuli such as stress, activity, and also brain injury.^39^ Based on this, it is not surprising that BDNF is thought to be of importance in the pathogenesis of depression. It has been observed that patients with psychiatric disorders and especially with depression often demonstrate reduced BDNF concentrations in their blood and brain, while antidepressant treatment can increase BDNF levels.^39^, ^41^ An underlying immunological pathway is suspected, as BDNF induces the expression of nuclear factor kB (NF‐kB) by binding to the tyrosine kinase B receptor (TrkB) and is thus involved in innate and adaptive immune response. As growing evidence supports a major role for NF‐kB in oncogenesis, BDNF has also been further investigated in the context of tumor disease. It is upregulated in various kinds of cancer and associated with tumor growth and metastasis.^42^, ^43^ An overexpression of TrkB and BDNF is linked to poor prognosis in certain types of cancer, such as NSCLC.^43^ Thus, unlike MCP‐1, the significance of BDNF as a biomarker for the occurrence of nAE must be rather doubted. Instead, the concentration differences at baseline might more likely be due to the tumor disease of the patients. This needs to be verified in a larger cohort.

Although the study prospectively investigated neurotoxicity under ICI therapy in detail and has impact on the therapeutic landscape by identifying a potential pretherapeutic biomarker, the evidence of the presented analyses is limited by their small sample size and needs to be confirmed in another study.

## 5 CONCLUSION

Neurological adverse events were found more frequently in our cohort than previously reported, sometimes reaching severe grades, as well as causing ICI treatment cessation. Here for the first time, sNFL was shown to increase significantly during the occurrence of nAE, confirming a mechanism of neurotoxicity. MCP‐1 and—with limitations—also BDNF were already elevated before therapy and might indicate an increased risk for the development of nAE and therefore potentially qualify as predictive biomarkers for nAE. Ultimately, those markers could reflect the first identified class‐specific biopredictors of ICI‐associated nAE, which is of particular interest also in other oncological contexts, such as in the prediction of chimeric antigen receptor (CAR)‐T cell‐induced neurotoxicity.

## AUTHOR CONTRIBUTIONS

**Nora Möhn:** Conceptualization (equal); funding acquisition (equal); project administration (equal); supervision (equal); writing – original draft (lead). **Susann Mahjoub:** Data curation (lead); investigation (lead); writing – original draft (supporting). **Laura Duzzi:** Investigation (supporting); writing – review and editing (equal). **Emily Narten:** Data curation (supporting); investigation (supporting); writing – review and editing (equal). **Lea Grote‐Levi:** Investigation (supporting); writing – review and editing (equal). **Gudrun Körner:** Data curation (supporting); writing – review and editing (equal). **Tabea Seeliger:** Investigation (supporting); writing – review and editing (equal). **Gernot Beutel:** Funding acquisition (equal); project administration (equal); resources (equal); writing – review and editing (equal). **Benjamin‐Alexander Bollmann:** Resources (equal); writing – review and editing (equal). **Thomas Wirth:** Resources (equal); writing – review and editing (equal). **André Huss:** Data curation (equal); resources (equal); writing – review and editing (equal). **Hayrettin Tumani:** Data curation (equal); resources (equal); writing – review and editing (equal). **Imke Grimmelmann:** Funding acquisition (equal); project administration (equal); resources (equal); supervision (equal); writing – review and editing (equal). **Ralf Gutzmer:** Funding acquisition (equal); project administration (equal); resources (equal); supervision (equal); writing – review and editing (equal). **Philipp Ivanyi:** Funding acquisition (equal); project administration (equal); resources (equal); supervision (equal); writing – review and editing (lead). **Thomas Skripuletz:** Conceptualization (equal); funding acquisition (equal); project administration (equal); supervision (equal); writing – review and editing (lead).

## FUNDING INFORMATION

The members of the ICOG‐study group received research funding from Bristol‐Myers Squibb Foundation for Immuno‐Oncology (FA 19‐010) and Claudia von Schilling Foundation for Breast Cancer Research.

## CONFLICT OF INTEREST STATEMENT

All authors declare that they have no competing financial interests or personal relationships that could have appeared to influence the work reported in this paper.

## INSTITUTIONAL REVIEW BOARD STATEMENT

The study protocol was approved by the local ethics committee at Hannover Medical School (No. 8685_BO_K2019) following the Declaration of Helsinki.

## INFORMED CONSENT STATEMENT

Written informed consent was obtained from all patients involved in the study.

## Supporting information


Data S1:

## Data Availability

All data relevant to the study are included in the article and uploaded as supplementary material. Further data can be made available upon reasonable request.
