# Supplementary material for: Monocyte chemoattractant protein 1 as a potential biomarker for immune checkpoint inhibitor‐associated neurotoxicity
Source: Cancer Med. 2023 Feb 16;12(8):9373–83. doi: 10.1002/cam4.5695 (PMC10166892; doi:10.1002/cam4.5695)
Supplement: Supplementary file 1 — Data S1: [file CAM4-12-9373-s001.pdf]

Supplemental material

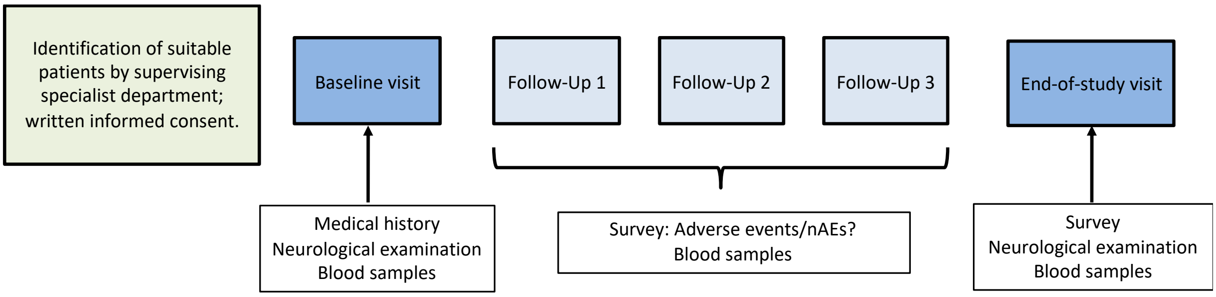

Supplemental figure 1: Study design and procedure. End-of-study visit is performed 6 months after baseline examination. Interval between the individual follow-up visits is 3-4 weeks. nAE: neurological adverse event.

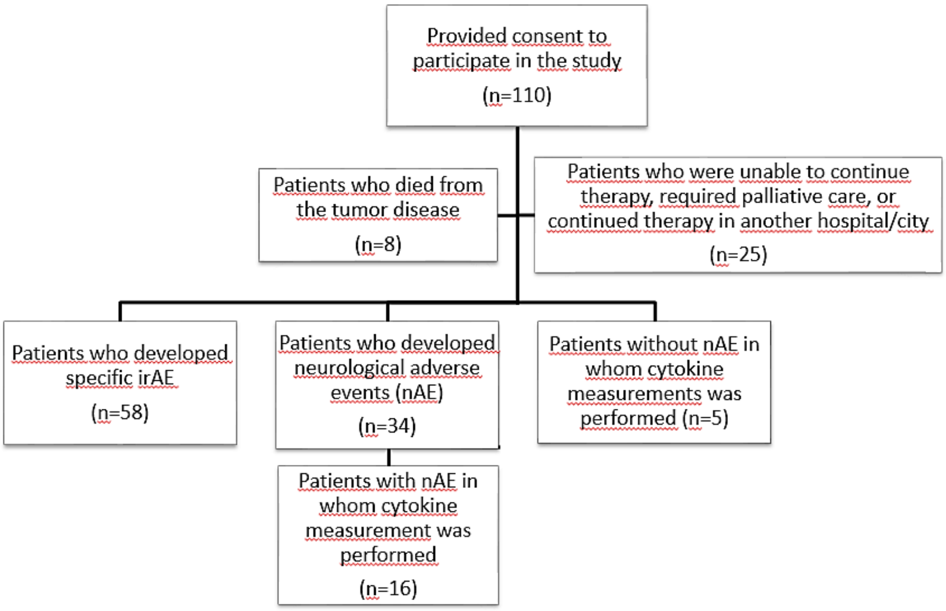

Supplemental figure 2: Consort figure. irAE: immune-related adverse events, nAE: neurological adverse events. 77 patients (90.6%) were still alive at time of last follow-up EOS. Eight patients (9.4%) died from the tumor disease during study conduction, while 25 patients (22.7%) discontinued the study early because they were unable to continue therapy, required palliative care, or continued therapy in another hospital/city.

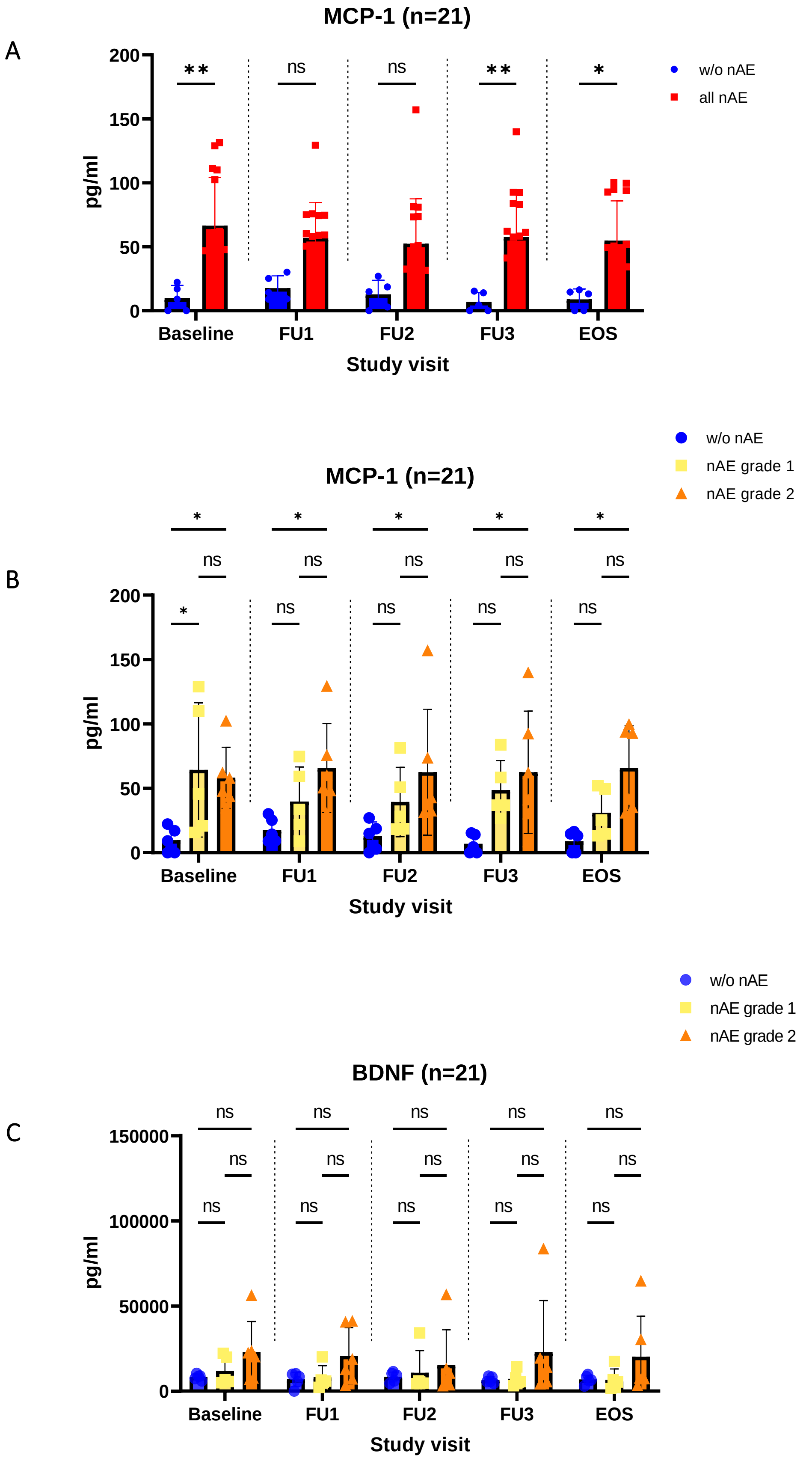

Supplemental figure 3: Cytokine measurement in patients with and without ICI-associated neurotoxicity. A) Serum monocyte chemoattractant protein 1 (MCP-1) concentrations, pooled comparison of all nAE(+) patients and patients without nAE. B) MCP-1 serum levels, comparison of patients without nAE

and those with nAE grade 1 and nAE grade 2. C) Serum levels of brain derived neurotrophic factor (BDNF), comparison of patients without nAE and those with nAE grade 1 and nAE grade 2. EOS: end-of-study, FU: follow-up, nAE: neurological adverse events, ns: not significant. ** p<0.01, * p<0.05.

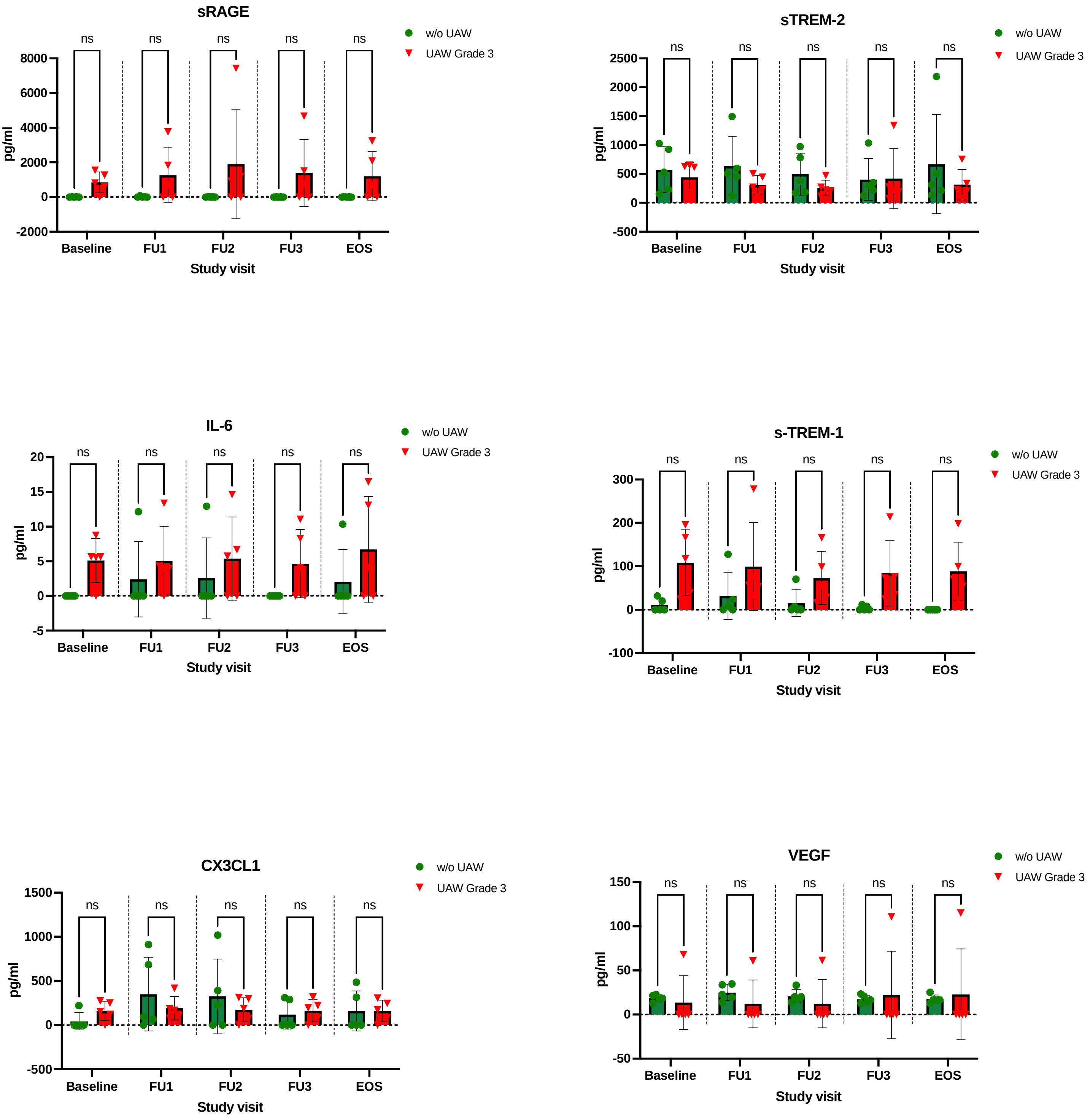

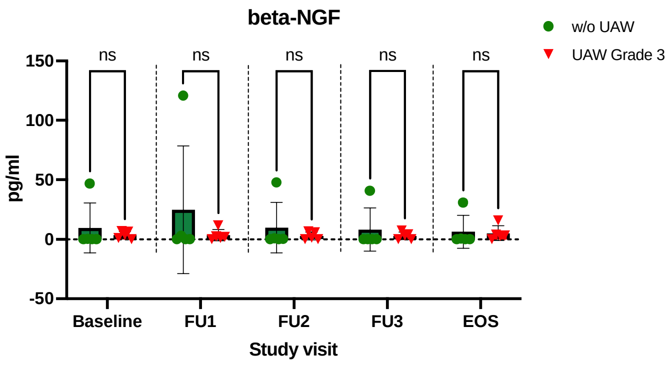

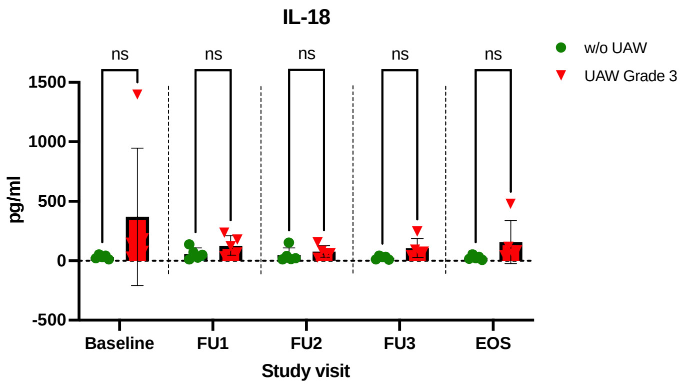

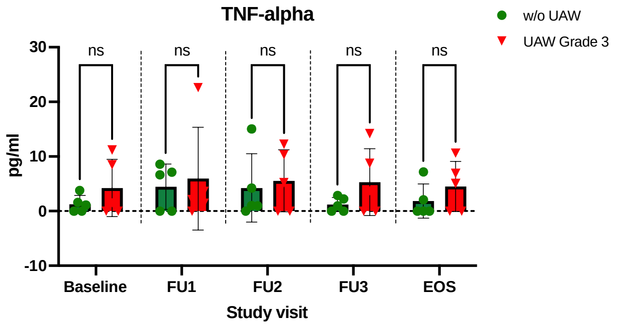

Supplemental figure 4: Presentation of all cytokines for which no significant difference was found in patients with and without higher-grade nAE. CX3CL: cytokine-induced neutrophil chemoattractant type-1, EOS: end-of-study, FU: follow-up, IL: interleukin, nAE: neurological adverse events, NGF: nerve growth factor, ns: not significant, sRAGE: soluble receptor for advanced glycation end products, TGF: transforming growth factor, TNF: tumor necrosis factor, TREM: triggering receptor expressed on myeloid cells, VEGF: vascular endothelial growth factor.

Supplemental table 1: Characteristics of “serum-subgroup”

Patient Nr	Age at ICI start	Sex	Underlying tumor disease	Nr of systemic therapies prior to ICI	Relevant comorbidities	Substance ICI	Termination of ICI due to AE (0= no, 1=yes)	Pausing ICI due to AE (0= no, 1=yes)	Hospitalisation due to AE (0= no, 1=yes)	Details irAE including nAE	Neurotoxicity CTCAE grade
1	74	f	Malignant melanoma	1	Cardiovascular	Nivolumab	0	0	0	Pruritus	0
2	56	m	Malignant melanoma	1	None	Nivolumab	0	0	0	None	0
3	30	m	Malignant melanoma	0	None	Nivolumab	0	0	0	Dysesthesia right scalp, pain left lower abdomen	0
4	52	m	Malignant melanoma	2	Cardiovascular	Nivolumab	0	0	0	Hyperthyroidism (asymptomatic)	0
5	66	m	Malignant melanoma	0	None	Nivolumab	0	0	0	None	0
6	68	f	Malignant melanoma	3	Cardiovascular	Nivolumab	0	0	0	**Tingling paresthesias,** pruritus, dry skin	1
7	66	f	Malignant melanoma	0	None	Nivolumab	0	0	0	**Mild tingling paresthesias**	1
8	29	m	Malignant melanoma	0	None	Nivolumab	0	0	0	**Hypesthesia in fingertips and toes,** diarrhea	1
9	48	f	Morbus Bowen	0	None	Cemiplimab	0	0	0	**Mild tingling paresthesias of both legs,** headache in the morning (spontaneous remission)	1
10	36	m	Malignant melanoma	1	None	Nivolumab/ Ipilimumab	0	0	0	**subjective decrease in strength, paresthesia right arm,** abdominal cramps, nausea, dry mouth, increased irritability	1

11	72	f	Malignant melanoma	0	Cardiovascular	Nivolumab	0	1	0	**Intense muscle pain (myopathy),** thyroiditis	2
12	75	m	Malignant melanoma	0	Cardiovascular_others	Nivolumab	1	1	0	**Pronounced lack of strength, balance disorders**	2
13	62	m	Malignant melanoma	0	Cardiovascular	Nivolumab	0	0	0	**Holocephalic headache with bulbar movement pain, intermittent paresthesias left hand, dizziness**	2
14	56	m	Malignant melanoma	1	Cardiovascular	Nivolumab/ Ipilimumab	0	0	0	**Myopathy (especially legs),** nausea, vomiting, diarrhea, hypothyroidism, pruritus	2
15	52	m	Renal cell carcinoma	0	Cardiovascular	Nivolumab/ Ipilimumab	0	0	0	**Hypesthesias, transient reduction in muscle strength**	2
16	60	m	Renal cell carcinoma	0	None	Nivolumab/ Ipilimumab	0	0	0	**Transient cramps and weakness of the left hand and legs,** dermatitis, mucositis °II	2
17	71	m	Malignant melanoma	1	None	Nivolumab	0	0	1	**Marked worsening of preexisting tremor and double vision (suspected autoimmune encephalitis),** dry skin	3
18	53	m	Malignant melanoma	0	None	Nivolumab	0	1	1	**Manifest polyneuropathy with leg paresis,** pruritic eczema	3
19	62	m	Tonsil carcinoma	0	Cardiovascular	Pembrolizumab	0	0	1	**Demyelinating sensorimotor polyneuropathy, recurrent absencelike conditions,** dermatitis III°	3
20	66	f	Malignant melanoma	2	None	Nivolumab/ Ipilimumab	0	0	1	**Giant cell arteritis with central retinal artery occlusion and left amaurosis**	3
21	58	f	Malignant melanoma	1	None	Nivolumab/ Ipilimumab	1	0	1	**Autoimmunencephalitis, demyelinating polyneuropathy,** lichenoid drug exanthema, vitiligo	3

Supplemental table 1: Characterization of patients in whom cytokines and neurofilaments were analyzed (so-called "serum subgroup"). Neurological adverse events are indicated in bold. AE: adverse event, CTCAE: common terminology criteria for adverse events, F: female, ICI: immune checkpoint inhibitor, irAE: immune related adverse event, m: male.
